# PvP01-DB: computational structural and functional characterization of soluble proteome of PvP01 strain of *Plasmodium vivax*

**DOI:** 10.1093/database/baaa036

**Published:** 2020-06-16

**Authors:** Ankita Singh, Rahul Kaushik, Dheeraj Kumar Chaurasia, Manpreet Singh, B Jayaram

**Affiliations:** 1 Supercomputing Facility for Bioinformatics & Computational Biology, Indian Institute of Technology Delhi, Hauz Khas, New Delhi, India, 110016; 2 Kusuma School of Biological Sciences, Indian Institute of Technology, Delhi, Hauz Khas, New Delhi, India, 110016; 3 Department of Chemistry, Indian Institute of Technology, Delhi, Hauz Khas, New Delhi, India, 110016; 4 Centre of Evolution and Medicine, Arizona State University, Life Sciences C, 427 East Tyler Mall, Tempe, AZ 85281, United States; 5 Laboratory for Structural Bioinformatics, Center for Biosystems Dynamics Research, RIKEN, 1-7-22 Suehiro-cho, Tsurumi-ku, Yokohama, Kanagawa 230-0045, Japan

**Keywords:** Malarial Infection, *Plasmodium vivax*, Computational Protein Databank, Functional Characterization; Protein Structure Prediction, Malarial Protein Targets

## Abstract

Despite *Plasmodium vivax* being the main offender in the majority of malarial infections, very little information is available about its adaptation and development in humans. Its capability for activating relapsing infections through its dormant liver stage and resistance to antimalarial drugs makes it as one of the major challenges in eradicating malaria. Noting the immediate necessity for the availability of a comprehensive and reliable structural and functional repository for *P. vivax* proteome, here we developed a web resource for the new reference genome, PvP01, furnishing information on sequence, structure, functions, active sites and metabolic pathways compiled and predicted using some of the state-of-the-art methods in respective fields. The PvP01 web resource comprises organized data on the soluble proteome consisting of 3664 proteins in blood and liver stages of malarial cycle. The current public resources represent only 163 proteins of soluble proteome of PvP01, with complete information about their molecular function, biological process and cellular components. Also, only 46 proteins of *P. vivax* have experimentally determined structures. In this milieu of extreme scarcity of structural and functional information, PvP01 web resource offers meticulously validated structures of 3664 soluble proteins. The sequence and structure-based functional characterization led to a quantum leap from 163 proteins available presently to whole soluble proteome offered through PvP01 web resource. We believe PvP01 web resource will serve the researchers in identifying novel protein drug targets and in accelerating the development of structure-based new drug candidates to combat malaria.

Database Availability: http://www.scfbio-iitd.res.in/PvP01

## Introduction

In 2018, World Health Organization (WHO) reported that malaria was endemic in 76 countries, with an estimated 219 million cases and 435 000 related deaths that occurred in the year 2017 (1). The reports moreover point out that progress is delayed after an unparalleled phase of achievement in global malaria control. Among the several parasitic species that cause human malaria, the neglected *Plasmodium vivax* is the most widespread of the malarial species (2, 3). More than one-third of the world’s population, nearly 2.5 billion people, is at risk of infection with *P. vivax* caused malaria (4). Despite the fact that *P. vivax* is the main offender in the majority of malarial infections outside Africa, very little information is available about its adaptation and development in humans (5). Its competence for instigating the relapsing infections through its dormant liver stage marks it as one of the major challenges in eradicating malaria. The emergence of resistance to antimalarial drugs, leading to an increase in severity and mortality, has emphasized the necessity to decrease the burden of *P. vivax* and warranting its eventual eradication (6, 7). Recent developments in the field of high-throughput sequencing technologies have led to the potential of performing whole-genome sequencing in a time- and cost-efficient manner (8, 9). These developments contributed immensely to exploring the genetic diversity among the Plasmodium species and laid ground for a better understanding of parasite biology and host–parasite interactions (6, 10–13).

The whole-genome studies when complemented with whole-proteome studies for structural and functional characterization may lead to a comprehensive understanding of *P. vivax* (14–17). Currently, two reference genomes of *P. vivax* strains are available, namely Salvador-I and PvP01. The Salvador-I reference is a monkey-adapted strain along with four other strains. Notably, the assembly of Salvador-I reference (prior to the curation) is highly fragmented and comprises more than 2500 scaffolds that restricted insights into the underlying biological mechanisms (18, 19). On the other hand, the PvP01 strain of *P. vivax,* isolated from a Papua Indonesian patient, resulted from a more comprehensive assembly using the high-depth Illumina sequencing, having only 226 scaffolds and better annotation as compared to its previous counterpart. Further, in PvP01, better annotation resulted in functional characterization of 58% core genes as compared to 38% in Salvador-I (19). The improved assembly and annotation of PvP01 qualify it as a very significant novel resource for a thorough study of *P. vivax-*caused malaria. Optimum utilization of the extensive genomic information of *P. vivax* available via its PvP01 strain may be complemented through structure-based drug discovery strategies. Unfortunately, there is no experimental structure available for any of the proteins corresponding to PvP01 for initiating structure-based drug design. Also, the *P. vivax* Sal-I proteome has only 1% of its protein structures experimentally addressed (20). Thus, development of a computational structural repository of *P. vivax* Sal-I proteome is attempted here to bridge the gap between sequence and structural information (21).

Considering the various stages involved in experimental methods, it is highly improbable to expect experimental structural information for the uncovered *P. vivax* proteome in the near future. A number of databases endeavor to provide insights on Plasmodium genus while offering massive data on annotated genomes, transcription level evidence, proteomics evidence, metabolic pathways and so on for various species of the malarial parasite (22–26). Despite the immediate urgency and availability of state of the art computational methods for prediction at protein structural and functional levels, there is not a single dedicated platform to study structural and functional information for PvP01, even after more than 3 years of its release. Taking into account the present scenario, we undertook the development of a web resource for delivering comprehensive structural and functional information of soluble proteome of *P. vivax* PvP01 strain. The web resource offered through a graphic user-friendly interface lends model structures filtered through an extensive quality assessment to ensure their reliability, functional characterization, information on potential ligand binding sites and comprehensive metabolic pathways developed with current state-of-the-art computational methods. We believe that the web resource will serve the researchers in identification as well as in designing and developing lead molecules that may eventually help in developing novel antimalarial drugs to combat *P. vivax* P01-caused malaria.

## Material and Methods

The development of the PvP01 web resource is performed at five different stages and at each stage various state of the art computational tools are employed to ensure reliability of predictions.

### Data sources

The whole proteome of the PvP01 strain of *P. vivax* was downloaded from PlasmoDB (22), which consisted of 6677 proteins. For the development of PvP01 web resource, all the membrane proteins were excluded. The soluble proteins were further screened for the presence of non-standard amino acid residues, presence of very small (less than 50 residues) and very large proteins (more than 1500 residues). The resultant set of proteins was further clustered at 100% sequence identity using standalone version of CD-HIT (27) to avoid redundancy. A list of filtered proteins is provided in the download section of database interface. These filters finally resulted in a dataset of 3664 non-membranous unique proteins of PvP01 strain of *P. vivax.* This dataset is named as soluble proteome of PvP01 strain of *P. vivax* hereafter. In PvP01 web resource, we furnish sequence, structural and functional information for the soluble proteome of PvP01 strain of *P. vivax.*

### Sequence-based information retrieval/prediction

For all the proteins in the soluble proteome of the PvP01 strain of *P. vivax*, a consensus secondary structure prediction approach was implemented by using standalone versions of PSIPRED (28), PSSPred (29) and SPIDER2 (30). Apart from secondary structure prediction, various physico-chemical features and homology-based features were calculated for individual proteins. Additionally, Structural Difficulty Index (SDI) (31) for all the proteins was calculated to assess the protein sequence modelability. A list of these features is provided in Supplementary Table S1. Details of these features are also provided in the help section of the PvP01 web resource.

### Protein structure prediction of soluble proteome of PvP01

The protein tertiary structure prediction for all the soluble proteins of the PvP01 strain of *P. vivax* was performed by implementing three different state of the art protein structure prediction software suites viz. I-TASSER (32), BhageerathH+ (33–36) and RaptorX (37). It may be noted that these software suites utilize different integrated approaches for protein structure prediction, namely *ab initio*, threading, homology modeling, profile-based fold recognition, etc., and thus bring completeness at structure prediction level. The availability, implementation and compute efficiency of these methods are summarized in Table 1 and explained further in Supplementary Table S2.

**Table 1:** Summary of various tools implemented in the development of the PvP01 web resource.

**Tool**	**Implementation stage**	**Availability**	**Compute**
SD Index	1° Structure information	http://scfbio-iitd.res.in/SDIndex	~0.1 hours
PSIPRED	2° Structure prediction	http://bioinf.cs.ucl.ac.uk/psipred/	~0.5 hours
PSSPred	2° Structure predication	https://zhanglab.ccmb.med.umich.edu	~1.5 hours
SPIDER3	2° Structure prediction	http://sparks-lab.org/server/SPIDER3	~2.0 hours
BhageerathH^+^	3° Structure prediction	http://scfbio-iitd.res.in/bhageerathH+	~24 hours
I-TASSER	3° Structure prediction	https://zhanglab.ccmb.med.umich.edu	~48 hours
RaptorX	3° Structure prediction	http://raptorx.uchicago.edu	~5 hours
GalaxyRefine	3° Structure refinement	http://galaxy.seoklab.org	~5 hours
ProTSAV	3° Structure assessment	http://scfbio-iitd.res.in/ProTSAV	~0.1 hours
ADDS	Ligand binding site	http://scfbio-iitd.res.in/ActiveSite	~0.1 hours
FPocket	Ligand binding site	http://fpocket.sourceforge.net	~0.1 hours
LIGSITE^CSC^	Ligand binding site	https://projects.biotec.tu-dresden.de	~0.1 hours
InterPro	Function annotation	https://www.ebi.ac.uk/interpro	~0.3 hours
SIFTER	Function annotation	https://sifter.berkeley.edu	~0.3 hours
LocTree3	Function annotation	https://rostlab.org/services/loctree3	~0.1 hours
ProBis	Function annotation	http://probis.cmm.ki.si	~1.5 hours
KEGG	Metabolic pathways	https://www.genome.jp/kegg	~1.5 hours
Total compute time required for an individual protein (~250 AA) on octa-core			~ 90 hours

The standalone versions (wherever available) of the listed tools are used and the compute efficiency provided here approximates the time required by the tool on an octa-core CPU machine for an individual protein of sequence length about 250 amino acid residues

### Protein structure refinement and quality assessment

For further improving the quality of predicted model structures, each structure was refined using a standalone version of GalaxyRefine (38). The GalaxyRefine-based protein structure refinement performs side chain rebuilding and repacking of input protein structure by implementing high probability rotamers, which is followed by global structure relaxation using molecular dynamics simulation. A combination of a physics-based energy function and harmonic restraint energy function is used for protein structure relaxation.

Also, to monitor the reliability of predicted model structures, an extensive quality assessment of all the structures was performed by implementing ProTSAV metaserver (39), which integrates 10 different tools of quality assessment. The details of these tools and ProTSAV-based protein structure quality assessment are provided in Supplementary Table S3. Based on the ProTSAV quality assessment, three model structures were selected for each protein i.e. the best model structure from I-TASSER, the best model structure from BhageerathH^+^ and the best model structure from RaptorX. At this stage, the PvP01 web resource has 10 992 model structures, three for each protein in the soluble proteome of PvP01 strain of *P. vivax.*

### Tertiary structure-based ligand-binding site prediction

For structure-based drug design, identification of potential ligand-binding sites in a target protein structure is a pre-requisite (40, 41). Considering the importance of identifying ligand binding sites, in the PvP01 web resource, we have provided potential ligand binding sites for all the 10 992 protein structures predicted and validated in the previous step. For identifying potential ligand binding sites, we implemented three different state-of-the-art methods, viz. F-Pocket (42), LigSite (43) and AADS (44). Details of various underlying approaches in these methods are provided in Supplementary Information (Table S2). The availability, implementation and compute efficiency of these methods are summarized in Table 1.

### Function annotation from sequence and structure

For comprehending various mechanisms at molecular level, a thorough knowledge of protein function is very fundamental. In PvP01 web resource, protein function annotation using four diverse tools is provided. These tools, namely SIFTER (45), LocTree3 (46), InterPro (47) and ProBiS (48), implement different strategies for performing function annotations of proteins. The details of various approaches implemented in individual tools are discussed in the Supplementary Information (Table S2). Additionally, the gene ontology terms for molecular functions, biological processes and cellular components are also extracted (wherever available) from UniProt database (49). The summary of availability, implementation and compute efficiency of these methods is provided in Table 1.

It is worth mentioning that the sequence, structure and functional information for an individual protein of about 250 amino acid residues furnished through the PvP01 web resource required about 90 compute hours on an octa-core CPU machine. The development of PvP01 web resource, accounting for more than 310 000 compute hours, is accelerated considerably with the help of parallel computing systems.

### Implementation and Application

#### PvP01 web resource architecture

A graphic user-friendly interface of the PvP01 web resource is developed through PHP, JavaScript and HTML5 implementation. Apache is used for providing webpage access services. The architecture safeguards the users with easy browsing through the PvP01 web resource. Additionally, the embedded Jsmol package (http://chemapps.stolaf.edu/jsmol) offers a better user experience for analyzing protein structures and ligand binding sites with a vast number of options.

**Figure 1:**
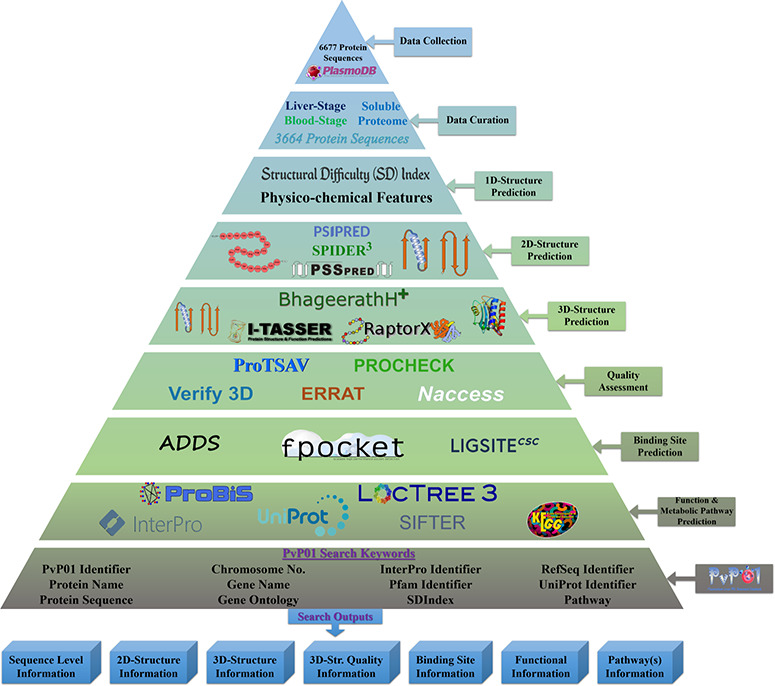
An overall workflow of PvP01 web resource development at different stages.

The overall architecture can be accessed through a wide range of web browsers. Figure 1 shows the overall workflow of the development of the PvP01 web resource at different stages.

#### Searching and browsing PvP01 web resource

The users can browse the PvP01 web resource containing information of the soluble proteome of the PvP01 strain of *P. vivax* by using a diverse range of keywords. For instance, UniProt Identifier, InterPro Identifier, Gene Ontology Terms, Protein Family Identifier, Protein Sequence, Gene Name, Protein Name, etc. are a few to mention. A list of most commonly used keywords is summarized in Table 2 for optimal use of this public web resource. Further details about the search keywords are provided in Supplementary Table S4.

**Table 2:** Summary of potential search keywords for browsing the PvP01 web resource efficiently

Search keyword	Example	Search keyword	Example
UniProt identifier	Q968V9	Protein name	Fructose-bisphosphate aldolase (EC 4.1.2.13)
Pfam identifier	PF00274	Gene name	PvP01_1262200
RefSeq identifier	YP_009325966	Protein sequence	MATGSE…. KKYVY
InterPro identifier	IPR029768	Chromosome number	12
PvP01 identifier	PvP01_1262200	Gene ontology term	GO:0003824

#### Automated database updating

PVP01 web resource has an option for updating the database as new information/structures become available. In addition to automatic periodic backend screening of protein databank for availability of new structural information, the user interface of the web resource offers an option to upload computationally modeled protein structure(s) (http://scfbio-iitd.res.in/PvP01/upload.php). In such cases, the user is needed to provide the UniProt identifier, its protein sequence and corresponding model structure. The PVP01 web resource will be able to compile the other additionally required information for the user’s updated protein at regular intervals.

#### Demonstration of stepwise browsing of PvP01 web resource

**Figure 2:**
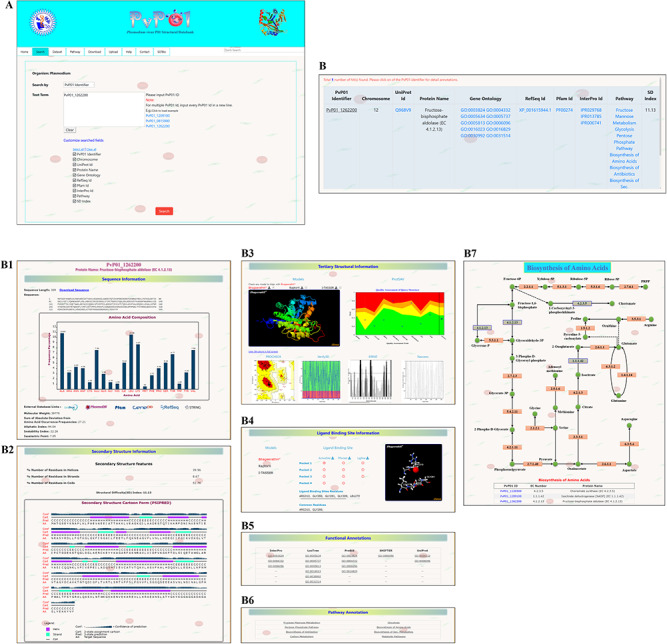
A depiction of different pieces of information provided in PvP01-DB about sequence, structure, ligand binding site, function annotations and metabolic pathways.

Here, we demonstrate a stepwise browsing of the PvP01 web resource using a gene ontology term (GO: 0003824) as a search keyword. Searching with a specific gene ontology term does offer a list of proteins that have that gene ontology term in their functional annotation. Users can navigate to the information of a specific protein by clicking on the link. On the protein-specific web page, users can browse various bits of information about its sequence, secondary structure, physico-chemical features, tertiary structure, quality assessment of structures, function annotation, potential ligand binding sites, etc. in an interactive manner. A pictorial representation of browsing of the web resource is provided in different panels in Figure 2.

## Results and Discussion

### Insights from SDI

**Figure 3:**
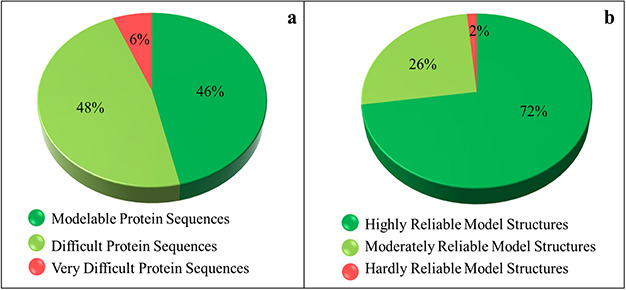
Sequence- and structure-based quantification of soluble proteome of PvP01 strain of *P. vivax.* (**a**) SDI-based characterization into Modelable, Difficult and Very Difficult regions of structural modelability. (**b**) Structure quality assessment of soluble proteome of PvP01 into highly reliable and hardly reliable regions of structural quality.

The SDI offers a quantification of the difficulty level of a protein sequence for modeling its tertiary structure. The soluble proteome of PvP01 strain of *P. vivax* could be characterized into modelable, difficult and very difficult on the basis of the various cutoffs of SDI. It is found that only 46% of soluble proteome (1702 proteins) seemed modelable for structure prediction and can be addressed using conventional homolog-based methods of structure prediction. This indicates that predicting reliable structures for the remaining 54% of the proteome is considerably challenging and requires substantial efforts to push them within the reliable limits of structure prediction. The SDI-based characterization of soluble proteome of PvP01 is shown in Figure 3(a).

### Insights from protein structure quality assessment

An extensive protein tertiary structure quality assessment of all the structures of soluble proteome of PvP01 is performed, using ProTSAV metaserver, to quantify them into highly reliable (within 3 Å rmsd), moderately reliable (3–5 Å rmsd) and hardly reliable (beyond 5 Å rmsd) regions of prediction. The quality assessment-based quantification of protein structures is shown in Figure 3(b). A comparison of Figure 3(a) and (b) indicates that we are able to push a considerable chunk of soluble proteome of PvP01 from difficult and very difficult regions of modelability to highly reliable regions of structural quality. It may be noted that majority of proteins have structures scoring in highly and moderately reliable regions. These structures can be directly used for various computational drug design and protein-protein interaction studies.

### Insights from computational protein functional characterization

**Figure 4:**
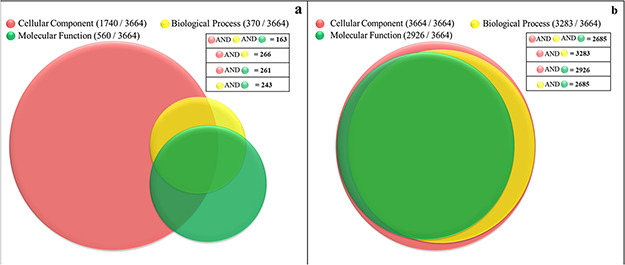
Functional characterization of soluble proteome of PvP01 strain of *P. vivax.* (a) Protein function information of the soluble proteome of PvP01 currently available in public resources. (b) Protein function information of soluble proteome of PvP01 furnished through the PvP01 web resource.

Currently, only 56% (2063 proteins) of the soluble proteome of PvP01 have gene ontology terms (GO terms) assigned as offered through UniProt and PlasmoDB. In gene ontology, there are three classes of GO term assignments, viz. molecular functions, biological processes and cellular components. Among these classes, molecular function assignment is the most informative as it directly indicates the molecular level activity of a gene product. The biological processes associated gene ontology terms reveal the overall biological process and the cellular component related terms provide insight about the cellular location for execution of the functions. Ideally, the complete information about molecular function, biological process and cellular component of a protein should be available for its comprehensive functional characterization. It is worth mentioning that only 163 out of the 3664 proteins of soluble proteome of PvP01 are assigned with gene ontology terms in UniProt and PlasmoDB with information about their molecular functions, biological processes and cellular components as shown in Figure 4(a). In PvP01 web resource, a broad level of computational functional characterization of soluble proteome of PvP01 is performed using several sequence-based and structure-based methods. With the help of the diverse methods, we are able to characterize all the proteins of the soluble proteome of PvP01. In terms of gene ontology terms assignment, we are able to push the boundary from 56% to 100% in all three classes. The additional gain of 44% could be achieved using InterPro-, SIFTER-, LocTree- and ProBis-based functional annotations. Importantly, for 2685 proteins of soluble proteome of PvP01, the web resource offers a fully comprehensive functional characterization through their molecular functions, biological processes and cellular components, which is a quantum leap from functional characterization of 163 proteins available presently. The functional characterization of soluble proteome of PvP01 offered via PvP01 web resource is depicted in Figure 4(b).

### Immediate application of PvP01 web resource

The structural and functional information furnished in the PvP01 web resource is coupled with the information derived from an extensive literature survey to development of metabolic pathways and identification of potential protein targets. The metabolic pathways for 223 proteins are developed (Supplementary Table S5) using the structural and functional information from PvP01 web resources and provided through the PvP01 web resource wherever available. Further, based on literature, 69 potential protein targets are identified from the soluble proteome of the PvP01 strain of *P. vivax.* The Food and Drug Administration (FDA), USA-approved library of drug molecules having non-human target proteins, adopted from Drug Bank (50, is screened against the potential target proteins. The target-ligand docking studies suggested 10 protein targets showing high binding affinity with some of the screened FDA-approved drug molecules. Keeping drug repurposing in mind, these potential protein targets are being subjected to molecular dynamics-based computational studies and their further experimental validation (work in progress).

## Conclusion

The improved assembly and better annotation of reference genome of *P. vivax* in its PvP01 strain over its previously reported Sal-I strain, makes PvP01 a better candidate to comprehend its various interactions and functionalities of use in development of anti-malarial drugs. Despite the availability of the whole-genome of PvP01, lack of reliable structural and functional information (experimental or computational) has delayed a thorough understanding of *P. vivax*-caused malaria and the development of efficient anti-malarial drugs. The web resource developed here is an earnest attempt to provide a comprehensive structural and functional repository about the soluble (non-membranous) proteome of PvP01. Only non-membrane proteins are considered in the present version of PvP01 web resource as the current state of the art methods for proteins structure prediction do not perform equally well for membrane proteins. However, contact-based methods seem to deliver improved results for membrane proteins, but require additional experimental data beyond sequences that are not currently available at proteome level. The pipeline used in the development of the resource implements consensus computational approaches to ensure reliability and completeness of the information provided. The availability of metabolic pathways and functional characterization obtained from sequence and structure-based state-of-the-art methods is of added advantage to the web resource. We believe that this web resource could be extremely useful to the research community for understanding and exploring the structural and functional aspects of *P. vivax* caused malaria. Furthermore, the structural information provided along with metabolic pathways could be helpful in identifying novel protein targets for initiating structure-based drug design.

## Supplementary Material

Supplementary_baaa036
