# Supplementary material for: PvP01-DB: computational structural and functional characterization of soluble proteome of PvP01 strain of Plasmodium vivax
Source: Database (Oxford). 2020 Jun 16;2020:baaa036. doi: 10.1093/database/baaa036 (PMC7296392; doi:10.1093/database/baaa036)
Supplement: Supplementary_baaa036 [file supplementary_baaa036.docx]

**Supplementary Information**

**PvP01-DB: Computational Structural and Functional Characterization of Soluble Proteome of PvP01 Strain of *Plasmodium vivax***

Ankita Singh^1, 4^, Rahul Kaushik^1, 5^, Dheeraj Kumar Chaurasia^1^, Manpreet Singh^1^, B. Jayaram^1, 2, 3, *^

^1^Supercomputing Facility for Bioinformatics & Computational Biology, Indian Institute of Technology Delhi, Hauz Khas, New Delhi, India

^2^Kusuma School of Biological Sciences, Indian Institute of Technology Delhi, Hauz Khas, New Delhi, India

^3^Department of Chemistry, Indian Institute of Technology Delhi, Hauz Khas, New Delhi, India

^4^Present Address: Centre of Evolution and Medicine, Arizona State University, Tempe, USA

^5^Present Address: Laboratory for Structural Bioinformatics, Center for Biosystems Dynamics Research, RIKEN, Yokohama, Japan

E-mail: [bjayaram@chemistry.iitd.ac.in](mailto:bjayaram@chemistry.iitd.ac.in); [ankita@scfbio-iitd.res.in](mailto:ankita@scfbio-iitd.res.in)

Website: [www.scfbio-iitd.res.in](http://www.scfbio-iitd.res.in)

**Supplementary Information**

**Table S1.** A List Sequence Based Features Provided in the PvP01 Web Resource.

| Sequence Length | Amino Acid Compositions |
| --- | --- |
| Protein Sequence | Secondary Structural Content |
| Isoelectric Point | Deviation for Occurrence Frequencies |
| Molecular Weight | Predicted Secondary Structure |
| Instability Index | Structural Difficulty Index |
| Aliphatic Index | External Database Links |

**Table S2.** Details of Tools Used in Development of PvP01 Web Resource.

| **Tool Name** | **Tool Description** |
| --- | --- |
| *SD Index* | Structural Difficulty (SD) Index provides insights into the possibility of a reliable tertiary structure prediction of a protein based on its physico-chemical, secondary structural and homology based feature calculation. |
| PSIPRED | PSIPRED is PSI-BALST based protein secondary structure prediction tool which implements neural networks on sequence profiles adopted from PSI-BLAST to predict the secondary structure of a protein sequence. |
| PSSPred | PSSPred is another Protein Secondary Structure Prediction tool based on simple neural network training algorithm. The prediction is integration of seven differently trained neural network learning models and profile data. |
| SPIDER3 | SPIDER3 is an iterative deep learning neural network based prediction software which implements sequence based local and non-local protein information in each iteration of training and prediction. |
| BhageerathH^+^ | BhageerathH^+^ is an integrated software suite for protein tertiary structure prediction of globular proteins. It implements *ab initio*, homology and hybrid approaches for structure prediction through its various modules. |
| I-TASSER | I-TASSER is an iterative threading, assembly and refinement approach based protein tertiary structure prediction software suite. The potential templates are identified by using multiple threading approach. |
| RaptorX | RaptorX is a statistical model which performs protein tertiary structure prediction via remote homolog detection and protein threading methods without performing conventional profile-profile matching. |
| GalaxyRefine | GalaxyRefine is a protein structural quality refinement tool which first rebuilds side chains of input protein structure, followed by its side-chain repacking and finally performs overall structure relaxation by molecular dynamics simulation to improve local and global structural quality. |
| ProTSAV | ProTSAV is a protein tertiary structure analysis and validation method which integrates 10 different individual tools of protein structural property calculation to perform a comprehensive and reliable quality assessment. |
| ADDS | The AADS methodology implements physicochemical features of functional groups lining the cavities on the protein surface for ligand binding site prediction. |
| FPocket | F-pocket predicts the potential ligand binding sites based on Voronoi partition and alpha sphere theory and the pocket ranking is performed via Partial Least Square fitting. |
| LIGSITE^CSC^ | LigSite captures surface-solvent-surface events via protein’s Connolly surfaces and identified pockets are ranked on the basis of the extent of conservation of surface residues involved. |
| InterPro | InterPro performs an extensive protein sequence analysis and classification via various prediction models assembled from different databases. |
| SIFTER | SIFTER offers a statistical approach using phylogenetic analysis for representing protein relationships and functional characterization. |
| LocTree3 | LocTree is a machine learning based hierarchical system for experimental localizations and function prediction of proteins. |
| ProBis | ProBis is a protein structure surface conservation based similar protein binding sites detection which assigns protein function depending on the extent of similarity of binding sites of target protein to the known proteins. |
| KEGG | The KEGG pathway map is a molecular interaction/reaction network diagram represented in terms of the KEGG orthology (KO) groups, so that experimental evidence in specific organisms can be generalized. |

**Table S3.** A summary of structural property assessment tools used as a part of ProTSAV for performing the quality assessment of predicted model structures in PvP01 web resource.

| PROCHECK | Procheck evaluates stereo chemical quality of a given protein structure by calculating deviation in the geometry of residues from their standard values which are derived from well-refined, high-resolution native structures. |
| --- | --- |
| ProSA | ProSA calculates overall protein structure quality score in terms of Z-score and performs statistical comparison with experimental protein structures. It utilizes knowledge-based Cα potentials of mean force to estimate model accuracy. |
| ProQ | ProQ is a neural-network based method which utilizes various structural features, such as frequency of atom–atom contacts, solvent accessible surface area, residue–residue contacts etc. and generates LG Score and MaxSub Score. |
| Verify-3D | Verify-3D compares 3D-1D profiles of a protein structure. The match of an atomic model (3D) is assessed with its own amino acid sequence (1D) and a quality score is predicted with parameters derived from database of experimental structures. |
| Naccess | Naccess calculates solvent exposed surface area of all atoms and residues with defined probe size. It helps in better insights on structure by comparing residue-wise surface area with experimental structure. |
| ERRAT | ERRAT distinguishes correctly modeled regions from incorrectly modeled regions in the protein structures by evaluating certain characteristic atomic interactions. |
| D2N | D2N is a random forest approach based machine learning tool which predicts the quality of a protein structure derived from different physico-chemical features of native protein structures. |
| dFIRE | dFIRE is an energy function which reports for pair-wise atomic interactions and dipole-dipole interactions among the amino acid residues. For a given protein structure it provides a set of free energy scores. |
| MolProbity | MolProbity assesses all-atom contacts, side chain clashes, and Ramachandran distribution of backbone dihedral angles (ɸ and Ψ) as the major structural parameters. |

**Table S4.** A summary of search keywords which can be used for a systematic and specific browsing of PvP01 web resource.

| **Search Keyword** | **Brief Description** |
| --- | --- |
| Protein Sequence | User can provide amino acid sequence in single letter code of desired protein. The output will result in a PvP01 identifier corresponding to input sequence. |
| Chromosome Number | The genome of *P. vivax* consists of 14 chromosomes and user can browse the database for proteins corresponding to any of the chromosomes by selecting chromosome number(s). |
| UniProt Identifier | **It is a unique identifier (6 or 10 letter alphanumeric string) assigned to each protein deposited in UniProt.** |
| Protein Name | User can explore the database with the help of protein names. |
| Gene Ontology (GO) | **The GO terms are unique accession numbers** of gene and gene product attributes across all species and can be used as search keywords in PvP01 web resource. |
| Protein Family or Pfam Identifier | Name of protein families or their Pfam identifiers can be used for exploring the databank. A Pfam identifier is a seven letter alphanumeric string. |
| InterPro Identifier | The InterPro database assigns each entry with a unique accession number in the form of an alphanumeric string starting with a prefix ‘IPR’ |
| RefSeq Identifier (RefSeq ID) | RefSeq Identifier is a unique identifier number for proteins in PvP01 we resource which refers to non-redundant and explicitly linked protein sequences. |
| PvP01 Identifier | PvP01 identifier is a 14 letter unique alphanumeric string assigned to all proteins of PvP01 strain of *P. vivax* by PlasmoDB. |

**Table S5.** A list of PvP01 identifier(s) for which the metabolic pathways information in provided in PvP01 web resource along with corresponding metabolic pathway(s) name.

| **PVP01 ID** | **Pathways** |
| --- | --- |
| PVP01_0116400 | Proteosome |
| PVP01_0117900 | Ribosome Biogenesis |
| PVP01_0118200 | Arginine Biosynthesis; Glutamate Metabolism; Terpenoid Backbone Biosynthesis; Metabolic Pathways |
| PVP01_0203400 | RNA Transport |
| PVP01_0208100 | Ribosome Biogenesis |
| PVP01_0210300 | Terpenoid Backbone Biosynthesis; Biosynthesis of Antibiotics; Biosynthesis of Sec. Metabolites; Metabolic Pathways |
| PVP01_0212000 | Mismatch Repair |
| PVP01_0212500 | N-Glycan Biosynthesis; Metabolic Pathways; Protein Processing in Endoplasmic Reticulum |
| PVP01_0303000 | Spliceosome |
| PVP01_0308000 | Malaria |
| PVP01_0308900 | DNA Replication; Homologous Recombination; Mismatch Repair; Nucleotide Excision Repair |
| PVP01_0309700 | RNADegradation |
| PVP01_0320000 | Ubiquinone Other Terpenoid-quinone Biosynthesis; Biosynthesis of Sec. Metabolites; Metabolic Pathways |
| PVP01_0320700 | Mismatch Repair |
| PVP01_0407900 | Metabolic Pathways; Oxidative Phosphorylation |
| PVP01_0410800 | Protein Processing in Endoplasmic Reticulum |
| PVP01_0413300 | N-Glycan Biosynthesis; Metabolic Pathways |
| PVP01_0414200 | SNARE Interactions in Vesicular Transport |
| PVP01_0415500 | Terpenoid Backbone Biosynthesis; Biosynthesis of Antibiotics; Biosynthesis of Sec. Metabolites; Metabolic Pathways |
| PVP01_0418300 | Malaria |
| PVP01_0418400 | Malaria |
| PVP01_0419100 | Nucleotide Excision Repair |
| PVP01_0421800 | Nucleotide Excision Repair |
| PVP01_0506200 | Aminoacyl-tRNA Biosynthesis |
| PVP01_0510700 | Lipoic Acid Metabolism; Metabolic Pathways |
| PVP01_0512500 | Phagosome; Protein Processing in Endoplasmic Reticulum; Protein Export |
| PVP01_0514100 | Glycerophospholipid Metabolism; Metabolic Pathways |
| PVP01_0514400 | RNA Degradation; Spliceosome |
| PVP01_0515500 | Ribosome Biogenesis |
| PVP01_0519200 | Metabolic Pathways |
| PVP01_0519400 | Glycine Serine and Threonine Metabolism; Glycolysis; Glyoxylate and Dicarboxylate Metabolism; Lysine Degradation; Propanoate Metabolism; Pyruvate Metabolism; TCA Cycle; Valine Leucine and Isoleucine Degradation; Biosynthesis of Antibiotics; Biosynthesis of Sec. Metabolites; Carbon Metabolism; Metabolic Pathways |
| PVP01_0526600 | Folate Biosynthesis; Pyrimidine Metabolism; Metabolic Pathways |
| PVP01_0529500 | Ribosome Biogenesis |
| PVP01_0530500 | Fatty Acid Elongation; Biosynthesis of Sec. Metabolites; Biosynthesis of Unsaturated Fatty Acids; Fatty Acid Metabolism; Metabolic Pathways |
| PVP01_0530800 | Phagosome |
| PVP01_0530900 | Ribosome |
| PVP01_0603100 | Proteosome |
| PVP01_0608000 | Biosynthesis of Antibiotics; Biosynthesis of Sec. Metabolites; Metbolic Pathways |
| PVP01_0612200 | Ribosome Biogenesis |
| PVP01_0612900 | Ribosome |
| PVP01_0617500 | Metabolic Pathways |
| PVP01_0617600 | Protein Processing in Endoplasmic Reticulum |
| PVP01_0619500 | TCA Cycle; Biosynthesis of Antibiotics; Biosynthesis of Sec. Metabolites; Carbon Metabolism; Metabolic Pathways; Oxidative Phosphorylation |
| PVP01_0621200 | Metabolic Pathways |
| PVP01_0622000 | Nucleotide Excision Repair |
| PVP01_0702100 | Phagosome |
| PVP01_0702800 | Protein Export |
| PVP01_0704900 | Phagosome |
| PVP01_0709100 | SNARE interactions in vesicular transport |
| PVP01_0709500 | Metabolic Pathways |
| PVP01_0710800 | Folate Biosynthesis; Metabolic Pathways |
| PVP01_0716000 | Spliceosome |
| PVP01_0717300 | Cysteine and Methionine Metabolism; Glutathione Metabolism; Metabolic Pathways |
| PVP01_0718000 | N-Glycan Biosynthesis; Metabolic Pathways; Protein Processing in Endoplasmic Reticulum |
| PVP01_0721400 | Biotin Metabolism; Fatty Acid Biosynthesis; Biosynthesis of Antibiotics; Fatty Acid Metabolism; Metabolic Pathways |
| PVP01_0722100 | Lipoic Acid Metabolism; Metabolic Pathways |
| PVP01_0722300 | Selenocompound Metabolism |
| PVP01_0723800 | Aminoacyl-tRNA Biosynthesis |
| PVP01_0724300 | RNA Transport; mRNA Surveillance |
| PVP01_0725200 | Metabolic Pathways |
| PVP01_0725800 | Pyruvate Metabolism; TCA Cycle; Biosynthesis of Antibiotics; Biosynthesis of Sec. Metabolites; Carbon Metabolism; Metabolic Pathways |
| PVP01_0726400 | Glycerophospholipid Metabolism; Biosynthesis of Sec. Metabolites; Metabolic Pathways |
| PVP01_0729100 | Glycerolipid Metabolism; Glycerophospholipid Metabolism; Biosynthesis of Sec. Metabolites; Metabolic Pathways |
| PVP01_0734000 | Metabolic Pathways |
| PVP01_0803300 | Ribosome |
| PVP01_0808400 | Phagosome |
| PVP01_0811300 | SNARE Interactions in Vesicular Transport |
| PVP01_0811500 | Proteosome |
| PVP01_0815100 | Biosynthesis of Sec. Metabolites; Metabolic Pathways |
| PVP01_0815300 | Aminoacyl-tRNA Biosynthesis |
| PVP01_0815900 | Glutathione Metabolism; Purine Metabolism; Pyrimidine Metabolism; Metabolic Pathways |
| PVP01_0817900 | Glycerolipid Metabolism; Metabolic Pathways |
| PVP01_0822500 | Ribosome Protein |
| PVP01_0826100 | SNARE Interactions in Vesicular Transport |
| PVP01_0829000 | Aminoacyl-tRNA Biosynthesis |
| PVP01_0831600 | Spliceosome; Ubiquitin mediated proteolysis |
| PVP01_0833100 | Ribosome Protein |
| PVP01_0833200 | Ribosome |
| PVP01_0833800 | Glycerophospholipid Metabolism; Biosynthesis of Sec. Metabolites |
| PVP01_0836000 | Endocytosis |
| PVP01_0837000 | Glycerolipid Metabolism; Glycerophospholipid Metabolism; Biosynthesis of Sec. Metabolites; Metabolic Pathways |
| PVP01_0904500 | Ribosome Biogenesis; RNA Polymerase |
| PVP01_0912500 | RNA Transport; Ribosome Biogenesis |
| PVP01_0917200 | N-Glycan Biosynthesis; Metabolic Pathways; Protein Processing in Endoplasmic Reticulum |
| PVP01_0918300 | RNA Transport; Ribosome Biogenesis |
| PVP01_0919400 | Endocytosis |
| PVP01_0920400 | Spliceosome |
| PVP01_0922700 | Metabolic Pathways |
| PVP01_0924000 | mRNA Surveillance |
| PVP01_0924600 | Protein Processing in Endoplasmic Reticulum |
| PVP01_0926700 | Aminoacyl-tRNA Biosynthesis |
| PVP01_0926900 | Ribosome |
| PVP01_0929400 | RNA Degradation |
| PVP01_0930200 | Base Excision Repair |
| PVP01_0930700 | Ribosome Protein |
| PVP01_0936800 | Fatty Acid Elongation; Biosynthesis of Sec. Metabolites; Biosynthesis of Unsaturated Fatty Acids; Fatty Acid Metabolism; Metabolic Pathways |
| PVP01_0938500 | Ribosome Protein |
| PVP01_1005100 | Spliceosome |
| PVP01_1005300 | Endocytosis |
| PVP01_1005600 | RNA Transport |
| PVP01_1013900 | Ribosome |
| PVP01_1014600 | Porphyrin and Chlorophyll Metabolism; Biosynthesis of Sec. Metabolites; Metabolic Pathways; Oxidative Phosphorylation |
| PVP01_1016500 | Amino Sugar and Nucleotide Sugar Metabolism; Galactose Metabolism; Biosynthesis of Antibiotics; Metabolic Pathways |
| PVP01_1017000 | Ribosome Protein |
| PVP01_1026300 | Protein Processing in Endoplasmic Reticulum |
| PVP01_1026900 | Ribosome Protein |
| PVP01_1031900 | Biosynthesis of Antibiotics; Biosynthesis of Sec. Metabolites; Metabolic Pathways |
| PVP01_1032800 | Peroxisome |
| PVP01_1104500 | Amino Sugar and Nucleotide Sugar Metabolism |
| PVP01_1104900 | Spliceosome |
| PVP01_1105200 | Glycerophospholipid Metabolism; Biosynthesis of Sec. Metabolites; Metabolic Pathways |
| PVP01_1112700 | RNA Polymerase |
| PVP01_1115600 | Ubiquitin Mediated Proteolysis |
| PVP01_1118200 | Nucleotide Excision Repair |
| PVP01_1121200 | Glycerophospholipid Metabolism; Biosynthesis of Sec. Metabolites; Metabolic Pathways |
| PVP01_1121600 | RNA Transport; Ribosome Biogenesis |
| PVP01_1121700 | Glycolysis; Glyoxylate and Dicarboxylate Metabolism; Propanoate Metabolism; Pyruvate Metabolism; Biosynthesis of Antibiotics; Biosynthesis of Sec. Metabolites; Carbon Metabolism; Metabolic Pathways |
| PVP01_1125500 | Amino Sugar and Nucleotide Sugar Metabolism; Fructose Mannose Metabolism; Galactose Metabolism; Glycolysis; Biosynthesis of Antibiotics; Biosynthesis of Sec. Metabolites; Carbon Metabolism; Metabolic Pathways |
| PVP01_1126500 | Biosynthesis of Amino Acids; Biosynthesis of Antibiotics; Biosynthesis of Sec. Metabolites; Metabolic Pathways |
| PVP01_1130100 | Protein Processing in Endoplasmic Reticulum |
| PVP01_1130600 | Metabolic Pathways |
| PVP01_1132300 | Pyruvate Metabolism; TCA Cycle; Biosynthesis of Antibiotics; Biosynthesis of Sec. Metabolites; Carbon Metabolism; Metabolic Pathways |
| PVP01_1133100 | Vitamin B6 Metabolism; Metabolic Pathways |
| PVP01_1134000 | Biotin Metabolism; Fatty Acid Biosynthesis; Biosynthesis of Antibiotics; Fatty Acid Metabolism; Metabolic Pathways |
| PVP01_1139500 | Glycerophospholipid Metabolism; Metabolic Pathways |
| PVP01_1141500 | Ubiquinone Other Terpenoid-quinone Biosynthesis; Biosynthesis of Sec. Metabolites; Metabolic Pathways |
| PVP01_1205600 | Aminoacyl-tRNA Biosynthesis; Porphyrin and Chlorophyll Metabolism; Biosynthesis of Sec. Metabolites; Metabolic Pathways |
| PVP01_1207700 | Homologous Recombination |
| PVP01_1209100 | Glutathione Metabolism; TCA Cycle; Biosynthesis of Amino Acids; Biosynthesis of Antibiotics; Biosynthesis of Sec. Metabolites; Carbon Metabolism; Metabolic Pathways; Peroxisome |
| PVP01_1210200 | Lipoic Acid Metabolism; Metabolic Pathways |
| PVP01_1210600 | Protein Processing in Endoplasmic Reticulum |
| PVP01_1211200 | Amino Sugar and Nucleotide Sugar Metabolism; Biosynthesis of Antibiotics; Metabolic Pathways |
| PVP01_1212000 | Glycolysis; Pyruvate Metabolism; TCA Cycle; Biosynthesis of Antibiotics; Biosynthesis of Sec. Metabolites; Carbon Metabolism; Metabolic Pathways |
| PVP01_1212300 | Malaria |
| PVP01_1213200 | Metabolic Pathways |
| PVP01_1216500 | Ribosome Protein |
| PVP01_1221800 | Aminoacyl-tRNA Biosynthesis |
| PVP01_1221900 | Ribosome Biogenesis |
| PVP01_1222100 | Base Excision Repair |
| PVP01_1222700 | SNARE Interactions in Vesicular Transport |
| PVP01_1223000 | Fatty Acid Elongation; Biosynthesis of Sec. Metabolites; Biosynthesis of Unsaturated Fatty Acids; Fatty Acid Metabolism; Metabolic Pathways |
| PVP01_1223300 | Protein Export |
| PVP01_1223800 | Metabolic Pathways |
| PVP01_1229800 | Folate Biosynthesis; Metabolic Pathways |
| PVP01_1229900 | SNARE Interactions in Vesicular Transport |
| PVP01_1231600 | Biotin Metabolism; Fatty Acid Biosynthesis; Fatty Acid Metabolism; Metabolic Pathways |
| PVP01_1238300 | Protein Processing in Endoplasmic Reticulum |
| PVP01_1239500 | Biosynthesis of Antibiotics; Biosynthesis of Sec. Metabolites; Metabolic Pathways |
| PVP01_1239700 | Base Excision Repair |
| PVP01_1241500 | RNA Transport |
| PVP01_1243100 | Nucleotide Excision Repair |
| PVP01_1245200 | Spliceosome |
| PVP01_1251000 | N-Glycan Biosynthesis |
| PVP01_1253000 | Spliceosome |
| PVP01_1254500 | SNARE Interactions in Vesicular Transport |
| PVP01_1260000 | Ribosome Protein |
| PVP01_1261900 | Aminoacyl-tRNA Biosynthesis |
| PVP01_1262200 | Fructose Mannose Metabolism; Glycolysis; Pentose Phosphate Pathway; Biosynthesis of Amino Acids; Biosynthesis of Antibiotics; Biosynthesis of Sec. Metabolites; Carbon Metabolism; Metabolic Pathways |
| PVP01_1263300 | Metabolic Pathways |
| PVP01_1263500 | RNADegradation |
| PVP01_1268500 | mRNA Surveillance |
| PVP01_1268900 | Protein Export; Protein Processing in Endoplasmic Reticulum |
| PVP01_1302500 | Protein Export |
| PVP01_1302900 | Porphyrin and Chlorophyll Metabolism |
| PVP01_1308100 | RNA Transport; mRNA Surveillance |
| PVP01_1309300 | SNARE Interactions in Vesicular Transport |
| PVP01_1310100 | Metabolic Pathways |
| PVP01_1310900 | DNA Replication |
| PVP01_1312600 | Purine Metabolism; Metabolic Pathways |
| PVP01_1314500 | Porphyrin and Chlorophyll Metabolism; Biosynthesis of Sec. Metabolites; Metabolic Pathways; Oxidative phosphorylation |
| PVP01_1315900 | Protein Export |
| PVP01_1319700 | Ribosome Protein |
| PVP01_1324700 | Ribosome Protein |
| PVP01_1327700 | Ribosome Protein |
| PVP01_1328500 | Aminoacyl-tRNA Biosynthesis |
| PVP01_1329100 | Glutathione Metabolism; Metabolic Pathways |
| PVP01_1334900 | Proteosome |
| PVP01_1338300 | RNA Transport |
| PVP01_1339000 | Glycerophospholipid Metabolism; Biosynthesis of Sec. Metabolites; Metabolic Pathways |
| PVP01_1346200 | Ribosome Protein |
| PVP01_1346800 | Purine Metabolism; Metabolic Pathways |
| PVP01_1347000 | Glycerophospholipid Metabolism; Metabolic Pathways |
| PVP01_1404100 | SNARE Interactions in Vesicular Transport |
| PVP01_1405400 | Protein Processing in Endoplasmic Reticulum |
| PVP01_1408500 | RNA Polymerase |
| PVP01_1409900 | RNADegradation |
| PVP01_1410300 | Spliceosome |
| PVP01_1410500 | Ribosome Biogenesis |
| PVP01_1411900 | Ribosome Protein |
| PVP01_1416500 | Glycerophospholipid Metabolism; Metabolic Pathways |
| PVP01_1416800 | RNA Transport |
| PVP01_1417700 | SNARE Interactions in Vesicular Transport |
| PVP01_1417900 | Spliceosome |
| PVP01_1418700 | Ribosome |
| PVP01_1421400 | Endocytosis |
| PVP01_1426400 | Ribosome |
| PVP01_1427500 | Spliceosome |
| PVP01_1429500 | Folate Biosynthesis; Metabolic Pathways |
| PVP01_1431400 | Glycerolipid Metabolism; Glycerophospholipid Metabolism; Biosynthesis of Sec. Metabolites; Metabolic Pathways |
| PVP01_1431600 | RNA Transport |
| PVP01_1432700 | RNA Transport |
| PVP01_1435400 | Malaria |
| PVP01_1442100 | Ribosome Protein |
| PVP01_1442800 | Porphyrin and Chlorophyll Metabolism |
| PVP01_1443100 | Spliceosome |
| PVP01_1444000 | Ubiquitin Mediated Proteolysis |
| PVP01_1449100 | Ribosome Biogenesis |
| PVP01_1450200 | Aminoacyl-tRNA Biosynthesis |
| PVP01_1450400 | Glycine Serine and Threonine Metabolism; Glycolysis; Glyoxylate and Dicarboxylate Metabolism; Lysine Degradation; Propanoate Metabolism; Pyruvate Metabolism; TCA Cycle; Valine Leucine and Isoleucine Degradation; Biosynthesis of Antibiotics; Biosynthesis of Sec. Metabolites; Carbon Metabolism; Metabolic Pathways |
| PVP01_1452400 | Base Excision Repair; DNA Replication; Nucleotide Excision Repair |
| PVP01_1455100 | Ubiquitin Mediated proteolysis |
| PVP01_1457200 | Ribosome Protein |
| PVP01_1458800 | DNA Replication; Mismatch Repair; Nucleotide Excision Repair |
| PVP01_1459800 | Ribosome Protein |
| PVP01_1460100 | SNARE Interactions in Vesicular Transport |
| PVP01_1460700 | RNA Transport |
| PVP01_1460900 | mRNA Survellance |
| PVP01_1461300 | Nucleotide Excision Repair |
| PVP01_1461700 | Endocytosis |
| PVP01_1462500 | Ribosome Protein |
| PVP01_1463900 | Protein Export |
| PVP01_1464400 | Metabolic Pathways |
| PVP01_1468800 | Aminoacyl-tRNA Biosynthesis |
